# Analysis of 13 *Alternaria* mycotoxins including modified forms in beer

**DOI:** 10.1007/s12550-021-00424-0

**Published:** 2021-03-05

**Authors:** Sophie Scheibenzuber, Fabian Dick, Stefan Asam, Michael Rychlik

**Affiliations:** Chair of Analytical Food Chemistry, Technical University of Munich, Freising, Germany

**Keywords:** *Alternaria* mycotoxins, Tenuazonic acid, Modified toxins, Stable isotope dilution assay, Beer

## Abstract

A multi-mycotoxin LC-MS/MS method was developed to quantify 13 free and modified *Alternaria* toxins in different beer types by applying a combination of stable-isotope dilution assays (SIDAs) and matrix-matched calibration. With limits of detection (LODs) between 0.03 µg/L (alternariol monomethyl ether, AME) and 5.48 µg/L (altenuene, ALT), limits of quantitation (LOQs) between 0.09 µg/L (AME) and 16.24 µg/L (ALT), and recoveries between 72 and 113%, we obtained a sensitive and reliable method, which also covers the emerging toxins alternariol-3-glucoside (AOH-3-G), alternariol-9-glucoside (AOH-9-G), alternariol monomethyl ether-3-glucoside (AME-3-G) and alternariol-3-sulfate (AOH-3-S) and alternariol monomethylether-3-sulfate (AME-3-S). Furthermore, 50 different beer samples were analyzed, showing no contamination with *Alternaria* toxins apart from tenuazonic acid (TeA) in concentrations between 0.69 µg/L and 16.5 µg/L. According to this study, the exposure towards TeA through beer consumption can be considered as relatively low, as the threshold of toxicological concern (TTC) value of 1500 ng/kg body weight per day might not be reached when consuming reasonable amounts of beer.

**Supplementary Information:**

The online version contains supplementary material available at 10.1007/s12550-021-00424-0.

## Introduction

With a per capita consumption of over 100 L of beer, Germany is among the top five countries with the highest beer consumption worldwide (World Health Organization 2018). Generally, an increase of beer consumption, on the one hand, and, on the other hand, a rising occurrence of mold growth on agricultural commodities due to unfavorable weather conditions can be observed. Both factors lead to concern in terms of mycotoxin exposure from beer consumption, as the main ingredients barley and wheat are often prone to fungal infection (Logrieco et al. 2009; Ostry 2008). Consequently, mycotoxin concentrations in beer should be monitored regularly to guarantee a safe product as toxins can be carried over from the grain into the final product. This was already shown for some *Fusarium* mycotoxins. Toxin concentrations generally increased during the malting step; however, the transfer rate into the beer was compound dependent (Habler et al. 2016, 2017). Although *Fusarium* mycotoxins are often considered the most critical risk factor during brewing, the malting process could also lead to production of *Alternaria* mycotoxins due to ideal growing conditions for *Alternaria* spp. (Bottalico and Logrieco 1998; MacLeod and Evans 2016). However, the behaviour of *Alternaria* toxins during the brewing process is still unclear, which makes further conclusions about mycotoxin transfer into the final product impossible.

**Fig. 1:**
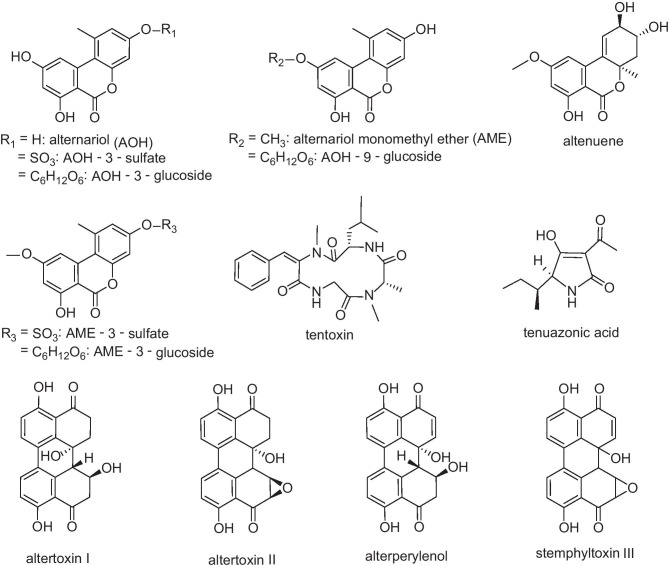
Chemical structures of 14 *Alternaria* mycotoxins including some modified forms. Due to instable standard solutions, the toxin stemphyltoxin III (STTXIII) was only qualitatively included in this study

In the field of *Alternaria* mycotoxins, the most frequently analyzed toxins are alternariol (AOH), alternariol monomethyl ether (AME), tenuazonic acid (TeA), altertoxins I and II (ATX I and ATX II), stemphyltoxin III (STTX III), tentoxin (TEN), altenuene (ALT) and alterperylenol (ALTP). However, also some modified forms of those toxins were detected recently and evoked increased attention in the last few years. These modified mycotoxins should also be included in analytical methods, because they might be able to release their parent toxin during digestion and therefore contribute to the total exposure towards mycotoxins (EFSA 2011). In the case of *Alternaria* toxins, some compounds are modified by the fungus itself, e.g. AOH-3-sulfate (AOH-3-S) and AME-3-sulfate (AME-3-S), while others are modified by the metabolism of the infected plant, e.g. AOH-3-glucoside (AOH-3-G), AOH-9-glucoside (AOH-9-G) and AME-3-glucoside (AME-3-G) (Soukup et al. 2016). Chemical structures of these compounds are shown in Fig. 1.

Until now, only a limited amount of data regarding *Alternaria* toxins in beer can be found in literature. Modified *Alternaria* toxins have not been analyzed in this matrix at all, yet. Furthermore, the presence of AOH, AME, TeA and TEN in various wine and juice samples shows the general ability of *Alternaria* toxins to be carried over through various food processing steps, which makes the analysis of beer particularly interesting (Broggi et al. 2013; Fan et al. 2016; Zwickel et al. 2016). In a study from 2010, Siegel et al. (2010) tested 43 beer samples for their TeA concentration and found an average TeA content of 11 µg/L, which seems reasonable as TeA is highly water soluble and might easily be carried through the brewing process. In another study conducted by Bauer et al. (2016), all 44 tested beer samples were positive for AOH with the highest concentration being 1.6 µg/L. Although these values seem to be relatively low, especially the AOH concentration can be critical when regarding the threshold of toxicological concern (TTC) value of 1.5 ng/kg body weight per day, which emphasises the importance of further studies in this field (EFSA 2011).

To evaluate the mycotoxin contamination in beers from German supermarkets and a local beer store, we aimed to develop a multi-mycotoxin liquid chromatography tandem mass spectrometry (LC-MS/MS) method to detect free and modified *Alternaria* toxins in different beer types. Besides the above-mentioned reports about the occurrence of TeA and AOH in beer (Siegel et al. 2010; Bauer et al. 2016), there is limited analytical data for this matrix available up to now. Also, the modified toxins AOH-3-G, AOH-9-G, AME-3-G, AOH-3-S and AME-3-S are only rarely included in analytical methods and were so far only determined in tomato products, cereal, fruit and vegetable juices as well as sunflower seed oil (Puntscher et al. 2018; Walravens et al. 2014, 2016). This study aims to get a better insight in *Alternaria* mycotoxin exposure through beer consumption and might give a first overview on toxins that can be carried over into beer during the brewing process.

## Materials and methods

### Chemicals and reference standards

Acetonitrile (ACN), cyclohexane and ammonia solution (25%) were purchased from VWR (Ismaning, Germany), methanol (MeOH) from Honeywell Riedel-de Haën (Seelze, Germany) and water from Th. Geyer (Renningen, Germany), all in analytical grade. Analytical standards for AOH, AME, ATXI, ATXII, STTXIII, ALTP, AOH-3-G, AOH-9-G and AME-3-G were isolated from fungal culture or synthesised at our chair as described previously (Gotthardt et al. 2020; Liu and Rychlik 2015; Scheibenzuber et al. 2020). TEN, ALT and TeA were bought from Merck (Darmstadt, Germany), and AOH-3-S and AME-3-S were isolated out of rice cultures of *Alternaria alternata*, as described in the following section. The labelled standards [^2^H_4_]-AOH, [^2^H_4_]-AME and [^13^C_6_,^15^ N]-TeA were synthesised in our laboratory as reported previously (Asam et al. 2009, 2011).

### Isolation of AOH-3-S and AME-3-S

To obtain the modified toxins AOH-3-S and AME-3-S, rice was inoculated with *A. alternata* as described by Gotthardt et al. (2020). Briefly, 25 g of parboiled rice and 15 mL of water were put into polycarbonate Erlenmeyer flasks and autoclaved (121 °C, 20 min). Spore suspensions of *A. alternata* isolated from potato leaves were diluted to 1.25 · 10^6^ spores/mL with a 0.5% Tween 20 solution, and 25 µL was added to the rice in the flasks. Cultivation took place at 26 °C and 110 rpm in a shaking water bath in the dark with exposition to artificial light for 30 min per day. After 7 days, rice cultures were thoroughly homogenised and extracted three times with 1 L of methanol/ethyl acetate (1/1; v/v) on a horizontal shaker (250 rpm) for 1h.

After decantation of the extracts and evaporation of solvents using a rotary evaporator (30 °C), the residue was dry loaded onto a silica column (125 g, Mesh70–230, pore size 60 Å, particle size 63–200 µm, Sigma-Aldrich, Steinheim, Germany), which was equilibrated with dichloromethane/methanol (9/1, v/v) beforehand. For elution of analytes, a gradient using 200 mL of different dichloromethane/methanol solutions (9/1; 7/1; 3/1; 1/1; 1/3; 1/7; 0/1; v/v) was applied, and fractions of 50 mL each were collected. All fractions were analysed by liquid chromatography with mass spectrometry in the full scan mode (LC-MS) using H_2_O/ACN (1/1; v/v) as mobile phase and a flow rate of 0.4 mL/min. Samples containing the desired analytes were combined, evaporated to dryness (30 °C) and dry loaded onto a second silica column for fractionation, using the same gradient and solvents as before. Again, all fractions containing AOH-3-S and AME-3-S were combined, evaporated to dryness (30 °C) and taken up in 10 mL H_2_O/ACN (1/1; v/v).

For the separation of AOH-3-S and AME-3-S, a semi-preparative HPLC system (LaChrom, D-7000, Merck, Germany, in cooperation with Hitachi Instruments Inc., San Jose, CA, USA) was used in combination with a Pro-Pack C18 column (150 × 10.0 mm, S-5 µm, 12 nm, YMC, YMC Europe GmbH, Dienslaken, Germany). Solvent A was ammoniumformate (5 mM in water), solvent B was 0.1% formic acid in acetonitrile, and the injection volume was 50 µL. The gradient was set as follows: 0–5 min 20% B, 5–25 min 20 to 65% B, 25–26 min 65 to 90% B, 26–39 min 90% B, 39–42 min 90 to 20% B and 42–50 min 20% B. Software for data analysis was the HPLC system manager (LaChrom, Merck, Germany, in cooperation with Hitachi Instruments Inc., USA, version 4.1).

The individual peaks for AOH-3-S and AME-3-S were identified based on their molecular mass using LC-MS full scans and collected from repetitive runs. For further identification, the isolated peaks were measured with LC-MS/MS, and the resulting fragment ions and retention times were compared with reference compounds we obtained from Hannes Puntscher (University of Vienna). For quantitation, samples were prepared for quantitative proton nuclear resonance spectroscopy (^1^H-qNMR) and measured on a Bruker AVIII system (400 MHz, Bruker, Rheinstetten, Germany) as described in Scheibenzuber et al. (2020) using the ^1^H signals of AOH for the quantitation of AOH-3-S, and the ^1^H signals of AME for the quantitation of AME-3-S.

### **Preparation of stock solutions**

After their synthesis, isolation or purchase, all toxins were identified with ^1^H-NMR, LC-MS and LC-MS/MS measurements and quantified by ^1^H-qNMR using the same parameters as described in Frank et al. (2014). Stock solutions of every analyte were prepared in concentrations between 1 and 100 µg/mL in acetonitrile (ATXI, ATXII, TEN, ALTP, ALT, AOH-3-G, AOH-9-G, AME-3-G, STTXIII) or methanol (AOH, AME, TeA, AOH-3-S, AME-3-S). For method validation and sample preparation, the stock solutions were further diluted and checked for purity using LC-MS full scans and LC-MS/MS. All standards were stored at −18 °C in the dark, and concentrations of diluted standards were checked regularly by UV spectrophotometric measurements (Genesys, 10S, UV–Vis spectrophotometer, Thermo Fisher Scientific, Madison, WI, USA) using precision cells made out of quartz glass (1 cm layer thickness, Hellma GmbH & Co. KG, Müllheim, Germany). Molar extinction coefficients were either obtained from literature (Cole et al. 2003; Fleck 2014; Visconti and Sibilia 1994; Scheibenzuber et al. 2020) or determined beforehand by identifying the UV absorption maximum via full scan and measuring three different dilutions in triplicate against the used solvent. The molar extinction coefficient ε (L mol^−1^ cm^−1^) was then calculated for each dilution using ε = (absorption · 1000)/(concentration [mmol/L] · 1 [cm]).

Although we initially developed the analytical method for 14 *Alternaria* mycotoxins, STTXIII was only available in low concentrations and showed a reduced stability in standard solutions, so this toxin was only included qualitatively in the final method.

### Samples

 Fifty different beer samples were bought in various supermarkets and a local beer store in Freising, Germany, during March and April 2019. Out of the 50 samples, nineteen were lager beers, eleven Pilsner, six bock beers, four wheat beers, two export beers and three craft beers (imported) as well as five other international beers.

### **Sample preparation**

Beer samples were degassed for at least 15 min and stored at −20 °C until further analysis. In triplicate, 5 mL of the degassed sample was measured into a 50-mL centrifuge tube and, for quantitation, spiked with 100 µL of a 0.1-µg/mL standard solution of [^13^C_6_,^15^N]-TeA, 100 µL of a 0.1-µg/mL standard solution of [^2^H_4_]-AOH and 100 µL of a 0.01-µg/mL standard solution of [^2^H_4_]-AME. To minimise matrix effects, 2.5 mL of cyclohexane was added to the sample, followed by shaking and centrifugation at 3220×*g* and 4 °C for 5 min. After removal of the cyclohexane phase, 15 mL of acetonitrile was added to the sample, which was then shaken vigorously to induce matrix precipitation. After centrifugation at 3220×*g* and 4 °C for another 5 min, the supernatant containing the extracted toxins was transferred into a 50-mL pear-shaped flask, while the precipitate was taken up in 10 mL ACN/H_2_O (84/16, v/v) and extracted for 10 min on a horizontal shaker. After extraction, the samples were centrifuged again at 3220×*g* and 4 °C for 5 min, and the supernatant was added to the first extract in the respective 50-mL pear-shape flask. Then, the solvent was removed using a rotary evaporator (40 °C). For further clean-up, the residue was taken up in 12 mL H_2_O (adjusted to pH 5.5 with formic acid) and then transferred onto Discovery® DSC-18 cartridges, which were preconditioned with 6 mL methanol and 6 mL H_2_O (pH 5.5), beforehand. After two washing steps, one with 6 mL H_2_O and one with 6 mL ACN/H_2_O (15/85, v/v), the toxins were eluted with 6 mL MeOH and 9 mL MeOH/25% ammonia solution (98/2, v/v). After rotary evaporation of the solvent, samples were taken up in 1 mL ACN/H_2_O (3/7, v/v), membrane filtered (0.22 µm) and stored at −18 °C until LC–MS/MS measurements.

### Multi-mycotoxin analysis via LC-MS/MS

 Chromatographic separation was performed on a Shimadzu Nexera X2 UHPLC system (Shimadzu, Kyoto, Japan). Separation of all analytes except TeA was done on a Hyperclone BDS C18 column (150 × 3.2 mm, 3 µm, 130 Å, Phenomenex, Aschaffenburg, Germany), and the binary gradient system was as follows: 0–2 min 10% B, 2–2.5 min 10 to 18% B, 2.5–10.5 min 18% B, 10.5–14 min 18 to 40% B, 14–20 min 40% B, 20–23 min 40 to 100% B, 23–25 min 100% B, 25–27 min 100 to 10% B and 27–32 min 10% B with a flow rate of 0.3 mL/min. Solvent A was water, solvent B acetonitrile. The column oven was tempered to 40 °C, and the injection volume was 10 µL. For the analysis of TeA, a Gemini-NX C18 column (150 × 4.6 mm, 3 µm, 110 Å, Phenomenex, Aschaffenburg, Germany) was used with 5 mM ammonium formate (adjusted to pH 9 with ammonia solution) as solvent A and methanol as solvent B, as published previously (Asam et al. 2013). Flow rate was set to 0.5 mL/min, and the oven temperature was 40 °C. The binary gradient system was 0–3 min 5% B, 5–8 min 5 to 100% B, 8–10 min 100% B, 10–13 min 100 to 5% B and 13–24 min 5% B. Both methods could be run in sequence as the system provided automated column switching and fourfold solvent selection for each pump.

**Fig. 2:**
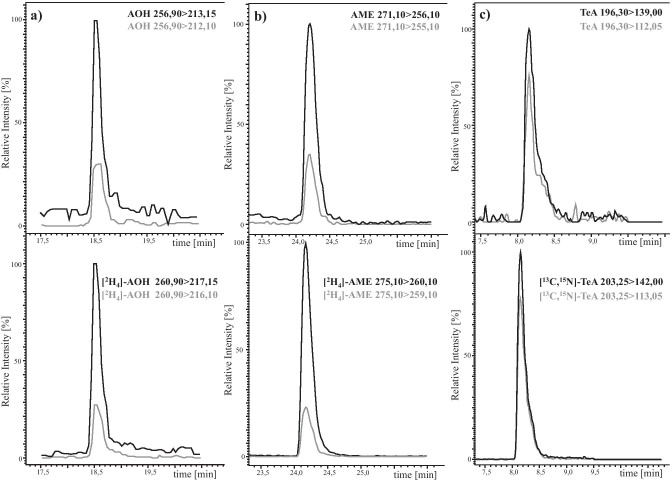
LC-MS/MS chromatograms of AOH (**a**), AME (**b**) and TeA (**c**), and their stable isotope-labelled analogues. The respective transition used for quantitation is shown in black and the transition used for confirmation in grey

**Fig. 3:**
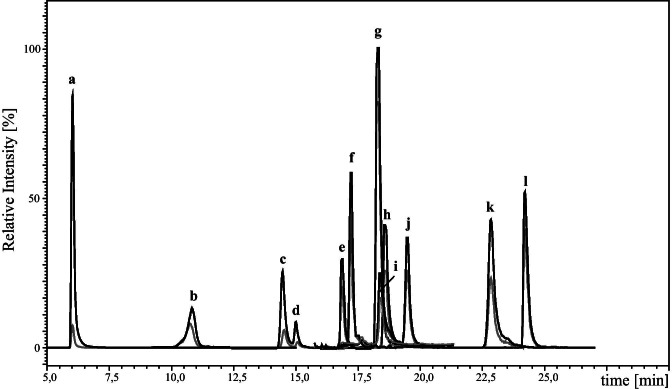
LC-MS/MS chromatogram of the 12 *Alternaria* mycotoxins AOH-3-S (**a**), AME-3-S (**b**), AOH-9-G (**c**), AOH-3-G (**d**), ALT (**e**), AME-3-G (**f**), ATXI (**g**), AOH (**h**), ALTP (**i**), TEN (**j**), ATXII (**k**) and AME (**l**). The chromatogram for TeA is included in Fig. 2

The LC was interfaced with a Shimadzu 8050 triple quadrupole mass spectrometer (Shimadzu Corporation, Kyoto, Japan). All measurements were conducted in the negative electrospray ionisation (ESI) mode. The ion source parameters were set as follows: interface temperature 300 °C, heat block temperature 400 °C, desolvation temperature 250 °C, interface voltage 4 kV, heating gas flow 10 L/min, drying gas flow 10 L/min, nebulizing gas flow 3 L/min, and collision-induced dissociation gas pressure 270 kPa. MS/MS measurements were operated in the multiple reaction monitoring (MRM) mode. All MS parameters were optimised by direct injection of each standard solution (0.01 to 1 µg/mL) into the ion source. The two most dominant mass transitions were included into the LC-MS/MS method with one serving as quantifier and one being used as qualifier for confirmation of peak identity. All final collision energies, voltages, fragment ions and retention times are listed in Table 1. Typical LC-MS/MS chromatograms are shown in Figs. 2 and 3.

**Table 1:** List of fragment ions and retention times (Rt) of the analyzed toxins and their corresponding optimised collision energies (CE) and voltages

Analyte	Rt (min)	Precursor ion *m/z*	Product ion *m/z*	Q1 pre-bias [V]	CE [V]	Q3 pre-bias [V]
AOH	18.63 ± 0.02	256.90	213.15	18	23	20
			212.10	48	29	38
[^2^H_4_]-AOH	18.60 ± 0.03	260.90	217.15	18	23	20
			216.10	48	29	38
AME	24.24 ± 0.01	271.10	256.10	20	23	24
			255.10	20	31	24
[^2^H_4_]-AME	24.22 ± 0.01	275.10	260.10	20	23	24
			259.10	20	31	24
ALT	16.79 ± 0.01	291.10	203.20	30	35	18
			248.15	24	27	14
ALTP	18.60 ± 0.03	349.10	261.20	26	30	26
			303.20	26	22	18
ATX I	18.23 ± 0.02	351.10	315.15	26	18	18
			297.15	26	28	18
ATX II	22.87 ± 0.03	349.10	313.20	16	18	20
			330.15	26	26	18
STTX III	23.38 ± 0.03	347.10	329.15	12	20	20
			301.10	16	35	30
TEN	19.43 ± 0.01	413.40	141.05	14	23	12
			214.25	14	26	20
TeA	8.16 ± 0.01	196.30	139.00	14	22	11
			112.05	22	26	20
[^13^C_6_,^15^ N]-TeA	18.16 ± 0.01	203.25	142.00	14	22	11
			113.05	22	26	20
AOH-3-S	6.05 ± 0.21	337.00	257.15	24	26	26
			213.15	24	39	20
AOH-3-G	14.95 ± 0.03	419.10	256.15	30	33	26
			228.20	30	45	12
AOH-9-G	14.22 ± 0.04	419.10	283.30	12	30	32
			256.15	18	35	28
AME-3-S	10.98 ± 0.2	351.20	271.20	12	23	26
			256.15	12	35	24
AME-3-G	17.38 ± 0.02	433.00	270.20	16	33	18
			227.10	12	54	20

For data acquisition and data analysis, the LabSolutions Software (Shimadzu, Kyoto, Japan) was used.

### Calibration and quantitation

 To obtain response curves for all analytes with a corresponding stable isotope-labelled standard, i.e., AOH, AME, and TeA, constant amounts of the labelled standards (S) [^2^H_4_]-AOH, [^2^H_4_]-AME, and [^13^C_6_,^15^N]-TeA were mixed with different amounts of the corresponding analytes (A) AOH, AME, and TeA in molar ratios between 0.01 and 100 (1:100, 1:50, 1:20, 1:10, 1:5, 1:2, 1:1, 2:1, 5:1, 10:1, 50:1, and 100:1). After LC-MS/MS measurements, response curves were obtained by plotting peak area ratios [A(A)/A(S)] against molar ratios [n(A)/n(S)]. Response functions were calculated by linear regression.

For analytes without a stable isotope-labelled standard, matrix-matched calibration was performed using a beer without the analytes of interest as a blank matrix. To obtain matrix-matched calibration curves, 8 to 10 matrix calibration points were prepared, ranging from 0.93 to 100 µg/L (TEN), from 0.62 to 10 µg/L (ATXI), from 1.75 to 10 µg/L (ATXII), from 2.84 to 10 µg/L (ALTP), from 16.1 to 100 µg/L (ALT), from 1.35 to 14 µg/L (AOH-3-G), from 1.3 to 14 µg/L (AOH-9-G), from 2.35 to 13 µg/L (AME-3-G), from 1.7 to 20 µg/L (AOH-3-S) and from 3.4 to 20 µg/L (AME-3-S). After LC-MS/MS measurements, matrix-matched calibration curves were obtained by plotting peak areas [A(A)] against concentration of the analytes [c(A)] and performing linear regression.

Linearity was confirmed for all analytes by applying Mandel’s fitting test (Mandel 1964).

The mycotoxin content in the beer samples was calculated either by the respective response curve or by matrix-matched calibration. To ensure correct values, two matrix calibration points were prepared with each sample work-up to compensate for intensity variability of the LC-MS/MS measurements.

### Method validation

LODs and LOQs. The limits of detection (LODs) and limits of quantitation (LOQs) were determined according to Vogelgesang and Hädrich (1998). The blank beer matrix was spiked at four different concentration levels with the unlabelled analytes AOH (0.4, 1.2, 2.6 and 4 µg/L), AME (0.05, 0.15, 0.33 and 0.5 µg/L), TeA (0.5, 1.5, 3.25 and 5 µg/L), TEN (0.2, 0.6, 1.3 and 2 µg/L), ATXI (0.3, 0.9, 1.95 and 3 µg/L), ATXII (0.2, 1, 2.5 and 4 µg/L), ALTP (0.6, 1.8, 3.9 and 6 µg/L), ALT (8, 24, 52 and 80 µg/L), AOH-3-G (0.3, 1.5, 3 and 4.5 µg/L), AOH-9-G (0.3, 1.5, 3 and 4.5 µg/L), AME-3-G (1.5, 5, 7.5 and 9 µg/L), AOH-3-S (0.2, 1.5, 3 and 5 µg/L) and AME-3-S (5, 7.5, 10 and 15 µg/L). After adding the isotope-labelled standards, the spiked blank samples were subjected sample preparation as described above and measured using LC-MS/MS. Every spiking level was prepared in triplicate.

### Precision

A beer sample naturally contaminated with TeA was spiked with the other toxins (0.8 µg/L AOH, 0.33 µg/L AME, 0.5 µg/L TEN, 0.9 µg/L ATXI, 4 µg/L ATXII, 1.8 µg/L ALTP, 24 µg/L ALT, 3 µg/L AOH-3-G, 3 µg/L AOH-9-G, 4 µg/L AME-3-G, 5 µg/L AOH-3-S and 7.5 µg/L AME-3-S) and used for intra-day (*n* = 3) and inter-day (*n* = 9, triplicate measurement every week within 3 weeks) precision measurements.

### Recovery

 Mycotoxin-free beer samples were spiked in triplicate with three different concentrations of AOH (1.2, 2.6 and 4 µg/L), AME (0.15, 0.33 and 0.5 µg/L), TeA (1.5, 3.25 and 5 µg/L), TEN (1.3, 2 and 2.5 µg/L), ATXI (0.90, 1.95 and 3 µg/L), ATXII (2.5, 4 and 5 µg/L), ALTP (3.9, 4.5 and 6 µg/L), ALT (24, 52 and 80 µg/L), AOH-3-G (1.5, 3 and 4 µg/L), AOH-9-G (1.5, 3 and 4.5 µg/L), AME-3-G (5, 7.5 and 9 µg/L), AOH-3-S (3, 5 and 7 µg/L) and AME-3-S (5, 7.5 and 10 µg/L) and analyzed after sample preparation. To obtain the recovery values, the ratio of detected and spiked contents was calculated.

## Results and discussion

### **Determination of UV molar extinction coefficients**

Molar extinction coefficients were determined for ALT and TEN in acetonitrile and for AOH-3-S, AME-3-S and TeA in methanol. The obtained values were used to regularly check standard concentrations of the reference compounds and to further characterise the modified toxins AOH-3-S and AME-3-S. The results are listed in Table 2.

**Table 2:** Absorption maxima and molar extinction coefficients of TEN, ALT, TeA, AOH-3-S and AME-3-S. Molar extinction coefficient values were rounded and are given as the mean value of three concentration levels measured three times in triplicate

Analyte	Absorption maxima (nm)	Concentration levels (µg/mL)	Solvent	Molar extinction coefficient ε (L mol^−1^ cm^−1^)
TEN	279 210	5.0/10.0/15.0	ACN	ε_279nm_ = 20,250 ± 1500
ALT	242 279	1.0/5.0/10.0	MeOH	ε_242nm_ = 28,700 ± 770
TeA	278 242	5.0/10.0/20.0	MeOH	ε_278nm_ = 13,900 ± 490
AOH-3-S	251 339	1.0/2.5/5.0	MeOH	ε_251nm_ = 48,300 ± 1800
AME-3-S	253 335	1.0/2.5/5.0	MeOH	ε_253nm_ = 55,400 ± 1500

### Sample preparation

 First, experiments showed that a dilute and shoot method did not deliver acceptable results in terms of sensitivity for this study. Consequently, different extraction and clean-up techniques were tested with spiked beer samples.

Matrix precipitation was a step commonly applied in beer analysis in literature, so we tested different volumes of ACN to find the most effective and economic method (Habler et al. 2017; Zachariasova et al. 2010). However, HPLC-UV studies revealed the presence of many matrix peaks, even after addition of high amounts of organic solvent, which required a further clean-up step using solid-phase extraction (SPE). Furthermore, AME tended to be bound by the precipitate, which could be solved by an additional extraction step of the residue. Unfortunately, direct use of the untreated beer for the SPE clean-up delivered good results for all toxins except the two very polar modified metabolites AOH-3-S and AME-3-S. These two analytes apparently did not bind to the solid phase either due to the alcohol content in the sample or other unfavorable interactions between analyte and sorbent material.

After further optimisation of the matrix precipitation and the SPE conditions, the matrix was clearly reduced when measured with HPLC-UV. However, when measured with LC-MS/MS, matrix effects were still observed, especially during the elution of the polar compounds AOH-3-S, AME-3-S, AOH-9-G and AOH-3-G, which negatively affected the LODs and LOQs. As those modified forms were never analyzed in beer before, we aimed to improve the analysis by testing an additional liquid-liquid extraction step. For this purpose, extraction with cyclohexane delivered the best results, even in small volumes. During this step, we observed a transition of low amounts of AME into the cyclohexane phase, which was decided to be negligible due to compensation by the SIDA used for this compound.

After combining all these steps, we obtained a sample preparation method that was highly efficient for the analysis of both free and modified *Alternaria* mycotoxins in beer samples and delivered reproducible values.

### Calibration and quantitation

Response functions were obtained for AOH, AME and TeA with their corresponding labelled analogues using linear regression. Linearity was confirmed by Mandel’s fitting test (Mandel 1964) between molar ratios n(A)/n(S) of 0.01–100 for all three analytes.

For the toxins without a labelled standard, matrix-calibration curves were generated by spiking a toxin-free beer sample in a concentration range starting from the LOQ of the respective toxin to at least ten times the LOQ. The linear range of the calibration was checked with Mandel’s fitting test (Mandel 1964) and the range reduced if necessary. Hence, linearity was confirmed from 0.93 to 100 µg/L for TEN, from 0.62 to 10 µg/L for ATXI, from 1.75 to 10 µg/L for ATXII, from 2.84 to 10 µg/L for ALTP, from 16.1 to 100 µg/L for ALT, from 1.35 to 14 µg/L for AOH-3-G, from 1.3 to 14 µg/L for AOH-9-G, from 2.35 to 13 µg/L for AME-3-G, from 1.7 to 20 µg/L for AOH-3-S and from 3.4 to 20 µg/L for AME-3-S.

## Method validation

The limits of detection (LODs) and limits of quantification (LOQs) were determined for every analyte in a toxin-free beer matrix as described by Vogelgesang and Hädrich (1998). The obtained LODs ranged from 0.03 µg/L (AME) to 5.48 µg/L (ALT), the LOQ values were between 0.09 µg/L (AME) and 16.24 µg/L (ALT). Detailed values for each toxin are listed in Table 3. Generally, the LODs and LOQs were low for all analytes determined by SIDA, i.e. AOH, AME and TeA. However, the values for the other toxins determined by matrix-matched calibration were also in a good range, which showed the good applicability of a matrix-matched calibration when stable isotope-labelled standards are not available. The only exception was ALT that has already been proven in literature to be most sensitively determined in the positive ESI mode. As this study used negative ESI mode, higher LOD and LOQ values had to be accepted for ALT in this multitoxin approach, because all other analytes were more sensitive in the negative mode than in the positive mode. However, the obtained values for ALT in this study are comparable with other methods using negative ESI mode (Walravens et al. 2014; Zwickel et al. 2016), but a measurement in the positive ESI mode or applying the polarity switching technique should be the methods of choice when focusing on ALT in the future (Prelle et al. 2013).

**Table 3:** Limits of detection (LODs), limits of quantitation (LOQs), recoveries, inter-injection precisions (*n* = 5), intra-day precisions (*n* = 3) and inter-day precisions (*n* = 9) for 13 *Alternaria* toxins in beer

Analyte	LOD (µg/L)	LOQ (µg/L)	Recovery (%)			RSD (%)		
			Level 1	Level 2	Level 3	Inter-injection	Intra-day	Inter-day
AOH	0.15	0.52	95 ± 4	95 ± 1	92 ± 3	2	5	5
AME	0.03	0.09	102 ± 1	100 ± 5	96 ± 3	2	2	3
TeA	0.15	0.46	87 ± 2	91 ± 1	86 ± 4	3	4	4
TEN	0.24	0.93	92 ± 5	105 ± 6	105 ± 8	3	7	8
ATX I	0.2	0.62	114 ± 4	111 ± 6	107 ± 7	4	6	9
ATX II	0.43	1.73	103 ± 7	98 ± 5	87 ± 2	5	5	8
ALTP	0.68	2.84	99 ± 3	110 ± 4	101 ± 7	4	8	8
ALT	5.48	16.24	107 ± 14	104 ± 8	106 ± 3	2	6	9
AOH-3-G	0.45	1.32	72 ± 3	94 ± 8	104 ± 8	2	5	9
AOH-9-G	0.32	1.28	101 ± 3	98 ± 10	101 ± 3	3	6	7
AME-3-G	0.72	2.32	113 ± 9	101 ± 2	103 ± 2	3	8	10
AOH-3-S	0.46	1.67	87 ± 7	84 ± 9	88 ± 5	4	5	8
AME-3-S	0.78	3.42	86 ± 1	96 ± 3	94 ± 5	4	9	10

The recovery of each analyte was determined by spiking blank beer matrix samples at three different concentration levels. According to theory, the spiking levels should comply with mycotoxin concentrations that are to be expected in naturally contaminated beer. However, as most of the analyzed 13 *Alternaria* toxins have never been found or analyzed in beer before, low or no toxin concentrations were expected. Consequently, the three spiking levels for recovery determination were chosen close to the LOQ. In this range, recoveries ranged from 72 to 113% (Table 3), which laid within the acceptable range of 70 to 120% (Vogelgesang and Hädrich 1998).

The precision of the developed method was calculated for each analyte as the relative standard deviation (RSD) of repeated measurements. Results are given in Table 3. To obtain the inter-injection precision, one spiked beer sample was repetitively measured (*n* = 5) with LC-MS/MS. Inter-injection RSD values ranged from 2 to 5%, which showed the stability of the used system. For the other RSD values, one sample was prepared in triplicate (intra-day precision) and again in triplicate every week within 3 weeks (inter-day precision). Intra-day precisions were 2 to 9% and inter-day precisions lay between 3 and 10%, which demonstrated good precision of the developed method.

### Screening of 13 free and modified *Alternaria* toxins

All 50 beer samples were prepared according to the sample work-up procedure described above and then screened for all *Alternaria* toxins except TeA using the LC-MS/MS multi-method. Measurements revealed that no free and modified toxins were present in any sample above their limit of detection, which was quite surprising in terms of AOH, as this toxin has been found in many beer samples before (Bauer et al. 2016; Prelle et al. 2013). All other analyzed toxins have also been found only rarely or not at all in previous studies (Prelle et al. 2013). Thus, it might be concluded that they were either not present in the raw material or their concentration decreased during the brewing process until below their limit of detection. However, this question must be addressed in further studies as data on *Alternaria* toxins in beer are only rarely available, especially for ALTP, ATXI, ATXII, ALT and TEN. As the focus of many mycotoxin multi-methods in cereal products including beer is not on *Alternaria* but on *Fusarium* toxins, mostly only the most prominent toxins AOH, AME and TeA were included into those methods, and data about other *Alternaria* toxins are missing. Also, modified forms of AOH and AME have never been analyzed before in this matrix.

In an Italian study, Prelle et al. (2013) found AOH in several beer samples from 2012 in a concentration range of 6.04–23.2 µg/L using LC–MS/MS with atmospheric pressure chemical ionisation (APCI), while Bauer et al. (2016) found lower amounts of AOH with concentrations ranging between 0.23 and 1.6 µg/L using enzyme-linked immunosorbent assay (ELISA). The difference between our results and the high AOH concentrations from Italy (Prelle et al. 2013) could partly be a result of the German purity law, which is obligatory for all beers produced in Germany and only allows few ingredients for beer production. Consequently, toxin levels can be higher when other ingredients, e.g. rice or maize, are used for the brewing process, which might elevate the total mycotoxin content in the final product. However, some foreign beers were also analyzed in this study and no AOH could be detected in those samples either.

Although Bauer et al. (2016) only found low amounts of AOH, they could detect it in every sample with their ELISA test with a LOD of 0.18 µg/L. Surprisingly, in our study, no beer was contaminated with AOH above the LOD of 0.15 µg/L. However, the spring and summer of 2018 was generally quite dry in Germany, which assumingly did not promote *Alternaria* spp. growth like in the years before. Therefore, the mycotoxin contamination of the 2018 harvest should have been relatively low, which could explain the divergent results from our study compared with the other studies. Also, Puangkham et al. (2017) analyzed 100 beer samples from Thailand and obtained similar results compared with our study, which again suggests that the mycotoxin concentration in beer can vary between different countries and different harvest or production years.

### Quantitation of TeA using stable-isotope dilution assay

 As expected, TeA was found in almost every sample, i.e. in 48 out of 50 beers, and in comparable amounts with a previous study (Siegel et al. 2010). Only two beers from Ireland were completely free of *Alternaria* mycotoxins, and all others contained TeA in concentrations between 0.69 ± 0.06 µg/L and 16.5 ± 2.2 µg/L. All beer samples were sorted into groups according to their beer type, and the average group mean value was calculated. An overview of the obtained results is shown in Table 4, and a detailed list of TeA concentrations can be found in the Supplementary Information.

**Table 4:** Summary of TeA concentrations in 50 beer samples, sorted into groups according to their beer type. Average concentrations of each sample were determined as mean value of three replicates and double injections

Group	Sample number	Samples containing TeA	Average TeA concentration (µg/L)	Highest TeA concentration (µg/L)
Pilsener beer	11	11	4.28 ± 3.00	8.87 ± 0.43
Lager beer	19	19	5.82 ± 3.18	12.21 ± 0.38
Wheat beer	4	4	5.72 ± 2.62	8.74 ± 0.75
Bock beer	6	6	11.35 ± 3.91	16.5 ± 2.2
Craft beer	3	3	3.14 ± 1.24	3.88 ± 0.29
Export beer	2	2	4.85 ± 3.21	7.12 ± 0.28
International	5	3	6.34 ± 7.56	3.40 ± 0.25

In our study, more samples were tested positive for TeA than in the study by Siegel et al. (2010), in which less than 40% of the samples contained TeA. This could be due to the different LOD and LOQ values and different analytical approaches: by using a derivatisation step for the analysis of TeA, Siegel et al. (2010) obtained a LOD of 2 µg/L and a LOQ of 8 µg/L, which is higher than that in our study. Although derivatisation normally leads to a more sensitive method, we could improve our LOD and LOQ values by applying SIDA and using an almost tenfold sample volume. Therefore, we could also detect low TeA concentrations in samples, which would probably have been tested negative in Siegel et al. (2010).

The standard deviation in the group mean values (Table 4) shows that the variation within the groups is high, which indicates that the TeA content might depend more on the contamination of the raw material than on the beer type. Siegel et al. (2010) proposed that the TeA content is higher in bock beers due to the higher wort content in this beer type. At first sight, our study seems to confirm this assumption, as both the sample with the highest content of TeA was a bock beer and also the mean concentration of TeA was highest in this type of beers. However, this finding has to be interpreted carefully, as we observed similar high concentrations of TeA in some samples of lager beers, and the dataset for bock beers was much smaller than for larger beers. From a theoretical point of view, when a lager and a bock beer are brewed from the same malt as starting material, it is most likely that the bock beer has a higher TeA concentration due to the higher wort content. However, as the toxin content in barley as well as in malt is highly variable, this assumption cannot be verified when different starting materials are used. For that reason, we propose that TeA concentration in beer is mainly dependent on the contamination of raw material and only less dependent on the wort content.

In conclusion, TeA was found to be the only relevant *Alternaria* toxin in beer in this study. However, toxin concentrations were quite low with a maximum value of 16.5 ± 2.2 µg/L, which was found in a bock beer. Therefore, the TTC value for TeA, i.e. 1500 ng/kg body weight per day, will not be reached by drinking reasonable amounts of beer. When an adult person with 70 kg body weight is consuming 1 L per day of the highest contaminated beer in this study, this will yield a daily intake of 235 ng/kg body weight per day. Nevertheless, in combination with other foods contaminated with TeA, beer consumption could considerably contribute to the total intake of TeA.

## Supplementary Information

Below is the link to the electronic supplementary material.

Supplementary file1 (DOCX 24 KB)

## Data Availability

The datasets generated during and analysed during the current study are available from the corresponding author on reasonable request.
