# Supplementary material for: Analysis of 13 Alternaria mycotoxins including modified forms in beer
Source: Mycotoxin Res. 2021 Mar 5;37(2):149–59. doi: 10.1007/s12550-021-00424-0 (PMC8163686; doi:10.1007/s12550-021-00424-0)
Supplement: Supplementary file 1 — Supplementary file1 (DOCX 24 KB) [file 12550_2021_424_MOESM1_ESM.docx]

**Supplementary Information**

**Analysis of 13 *Alternaria* mycotoxins including modified forms in beer**

**Sophie Scheibenzuber, Fabian Dick, Stefan Asam*, Michael Rychlik**

Chair of Analytical Food Chemistry, Technical University of Munich, Freising, Germany

*Corresponding author:

Dr. Stefan Asam

Chair of Analytical Food Chemistry

Maximus-von-Imhof Forum 2

85354 Freising

Germany

[stefan.asam@tum.de](mailto:michael.rychlik@tum.de)

**TeA concentrations of each analyzed beer**

Table 1. TeA concentrations as mean value of three replicates and double injection, wort and alcohol content of the 50 analyzed beer samples, sorted into different beer types.

| Beer type | Alcohol content (% vol) | Wort (°P) | TeA concentration [µg/L] |
| --- | --- | --- | --- |
| Pilsener | 4.9 | 11.2 | 4.68 ± 0.37 |
| (n = 11) | 4.4 | 11.75 | 8.63 ± 1.01 |
|  | 4.9 | 11.0 | 8.87 ± 0.43 |
|  | 4.9 | 11.3 | 2.24 ± 0.16 |
|  | 4.9 | 11.0 | 5.54 ± 0.46 |
|  | 4.7 | 11.2 | 7.96 ± 0.62 |
|  | 4.8 | 11.2 | 0.94 ± 0.05 |
|  | 4.8 | 11.2 | 2.09 ± 0.09 |
|  | 4.8 | 11.6 | 2.11 ± 0.23 |
|  | 4.9 | 11.2 | 2.07 ± 0.28 |
|  | < 0.5 | 11.3 | 1.99 ± 0.08 |
| Lager Beer | 5.1 | 11.7 | 3.27 ± 0.23 |
| (n = 19) | 4.9 | 11.7 | 3.09 ± 0.25 |
|  | 4.9 | 11.5 | 2.34 ± 0.22 |
|  | 5.1 | 11.8 | 12.21 ± 0.38 |
|  | 5.2 | 11.5 | 5.34 ± 0.26 |
|  | 4.9 | 11.0 | 6.24 ± 0.29 |
|  | 5.4 | 12 | 3.24 ± 0.10 |
|  | 5.9 | 13.5 | 7.74 ± 0.26 |
|  | 4.7 | 11.5 | 4.60 ± 0.27 |
|  | 5.6 | 12.0 | 7.74 ± 0.16 |
|  | 4.9 | 11.2 | 0.93 ± 0.03 |
|  | 5.6 | 13.3 | 8.34 ± 1.01 |
|  | 4.9 | 11.0 | 9.87 ± 0,29 |
|  | 5.6 | 12.6 | 11.62 ± 0.24 |
|  | 5.5 | 12.5 | 5.43 ± 0.50 |
|  | 5.2 | 11.8 | 5.08 ±0.22 |
|  | 5.0 | 11.4 | 2.38 ± 0.17 |
|  | 5.6 | -* | 7.07 ± 0.26 |
|  | 5.1 | 12.5 | 2.42 ± 0.16 |
| Wheat Beer | 5.4 | 12.0 | 4.08 ± 0.33 |
| (n = 4) | 5.4 | 12.8 | 2.04 ± 0.14 |
|  | 5.0 | 11.8 | 4.35 ± 0.22 |
|  | 5.2 | 12.8 | 8.74 ± 0.75 |
| Bock Beer | 7.0 | 16.0 | 8.65 ± 0.69 |
| (n = 6) | 7.5 | 18 | 9.54 ± 0,87 |
|  | 8.0 | 17.5 | 15.80 ± 1.35 |
|  | 6.9 | 16.5 | 6.98 ± 0.53 |
|  | 7.4 | 18.3 | 16.5 ± 2.2 |
|  | 7.7 | 16.5 | 10.60 ± 7.73 |
| Craft Beer | 5.0 | 11.7 | 1.76 ± 0.18 |
| (n = 3) | 4.7 | 11.4 | 3.88 ± 0.29 |
|  | 6.0 | 14.0 | 3.83 ± 0.14 |
| Export Beer | 5.6 | 12.0 | 2.58 ± 0.14 |
| (n = 2) | 5.2 | 12.3 | 7.12 ± 0.28 |
| International | 5.0 | 11.9 | 14.93 ± 1.24 |
| (n = 5) | 4.2 | 11.9 | n.d. |
|  | 4.2 | 11.9 | n.d |
|  | 4.5 | 11.3 | 3.40 ± 0.25 |
|  | 6.6 | -* | 0.69 ± 0.06 |

n.d.: not detected; *: not available
